# Heterotopic calcification in a child presenting as acute on chronic myelopathy

**DOI:** 10.1007/s00381-025-06861-x

**Published:** 2025-06-10

**Authors:** Tyler Zeoli, Preethi Reddi, Reena Singh, Benjamin Greene, Michael C. Dewan

**Affiliations:** 1 Department of Neurological Surgery, Vanderbilt University Medical Center, Nashville, TN USA; 2 Medical College of Georgia, Augusta, GA USA; 3 Department of Pathology, Vanderbilt University Medical Center, Nashville, TN USA; 4 Department of Radiology, Vanderbilt University Medical Center, Nashville, TN USA; 5 Surgical Outcomes Center for Kids (SOCKs), Monroe Carell Jr. Children’s Hospital at Vanderbilt, Nashville, TN USA

**Keywords:** Pediatric spinal cord injury, Heterotopic calcification, Cervical spine, Heterotopic ossification, Central cord syndrome

## Abstract

**Purpose:**

Heterotopic calcification (HC) is a rarely reported pathology of aberrant bone deposition in extraskeletal tissue, most commonly outside of the central nervous system. While some of these findings may be incidental and asymptomatic, patients with symptomatic cord compression due to HC require consideration for expedited surgical intervention. We present the first described pediatric case of HC of the cervical spine causing spinal cord compression in a patient presenting with acute weakness and paresthesias following a minor trauma.

**Case report:**

An 11-year-old male with a history of long-standing spastic hemiplegia thought to be related to perinatal hypoxia presented acutely myelopathic after a minor trauma. Imaging revealed an extradural calcification of the upper cervical spine with severe spinal cord compression and a focal kyphotic deformity, considered most likely to represent a calcified meningioma, nerve sheath tumor, or heterotopic calcification. The patient was taken for decompression, mass resection, and laminoplasty, with final pathology revealing heterotopic calcification.

**Conclusion:**

While HC can develop after traumatic insult, or as late sequelae of spontaneous hemorrhage or infection, involvement of the cervical spine in a child has not previously been reported. In the setting of severe spinal cord compression with motor deficits, decompression and complete resection are safe and feasible. Histological analysis of HC will demonstrate a zonal arrangement of peripheral spindle cells/fibrous tissue, myxoid/cartilaginous tissue, and an inner core of ossification. Close attention should be paid in infancy when there may be an unclear diagnosis for weakness or spasticity without full imaging of the neuroaxis.

## Introduction

Heterotopic calcification (HC) is a pathology of aberrant bone deposition in extraskeletal tissue and remains rarely reported in the literature [1, 2]. The most common etiology stems from traumatic sequelae, followed by tumoral or genetic origins [3]. Varying levels of TGF-β, IL-1, IL-2, and leptin increase osteoblastic activity following traumatic insult leading to bone formation [4–8]. HC most commonly occurs surrounding joints and soft tissue, with no current reports of spinal cord involvement. Of note, while 10% of pediatric patients with traumatic spinal cord injury (SCI) can develop HC, this has been linked to progressive spasticity secondary to calcification of tissue outside of the central nervous system [8–10].

Other less commonly reported manifestations of atypical calcification in the spine include diffuse spinal dural calcifications in the setting of hyperparathyroidism and ligamentum flavum calcification [2–15]. While these findings may be incidental and asymptomatic, a patient with HC within the spinal canal can present with cord compression necessitating surgical decompression. Herein, we present a case of HC of the cervical spine causing spinal cord compression in a child with subtle underlying myelopathy presenting with acute weakness and paresthesias following minor trauma.

## Case report

### Presentation and perioperative management

An 11-year-old male with a history of right-sided spastic hemiparesis presented with central cord syndrome, including bilateral upper greater than lower extremity weakness and dysesthetic pain, after wrestling with an older sibling. Born at 36 weeks gestation via cesarean section, his childhood was notable for slow progression of motor milestones, left-sided preference for motor tasks, and right-sided spasticity, which was ultimately diagnosed as spastic cerebral palsy. He subsequently underwent extensive orthopaedic interventions to address the sequelae of presumed cerebral palsy, including serial bracing, femoral osteotomy, and tendon lengthening procedures. Imaging of the neural axis was never obtained, and no further workup was completed for his seemingly chronic underlying myelopathy.

During the index presentation to the emergency department, the dysesthetic pain resolved, but some numbness in his bilateral hands persisted. He was 4/5 strength in his right deltoid with a positive Hoffman sign. As was his baseline, he demonstrated ambulatory spastic quadriparesis with increased tone on the right side. A CT of the cervical spine demonstrated a large, calcified mass occupying the majority of the canal, causing focal kyphosis. MRI revealed a large extradural mass from C2 to C5 with heterogeneous enhancement, adjacent dural enhancement, and internal calcifications. There was displacement and severe compression of the spinal cord. No other lesions were identified throughout the neuroaxis.

### Surgical intervention

**Fig. 1:**
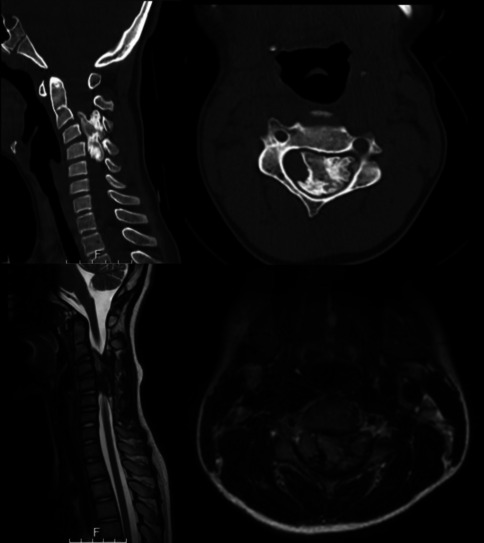
Preoperative non-contrast CT (above) and T2 MRI of cervical spine (sagittal and axial views) displaying a left eccentric calcified mass from C2 to C5 with severe spinal cord compression

**Fig. 2:**
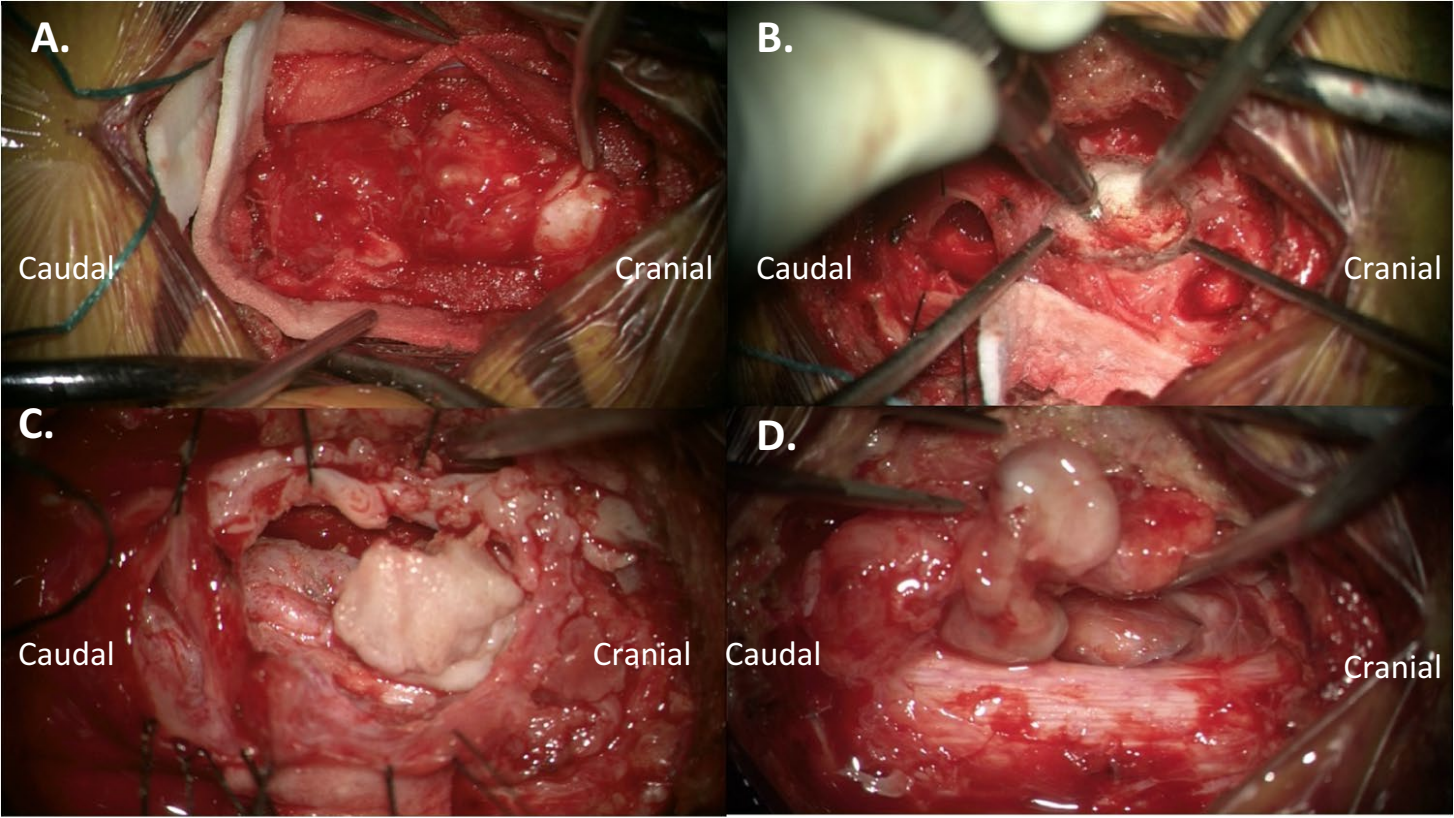
Intraoperative microscope images depicting (**A**) initial dissection visualizing pearly white ossified lesion at cranial portion; (**B**) utilization of burr for piecemeal debulking of lesions within the central canal; (**C**) mobilized lesion after debulking the cranial portion; and (**D**) re-expansion of the spinal cord along right side, with visualization of the capsular portion of the tumor adjacent to exiting C4 nerve root

**Fig. 3:**
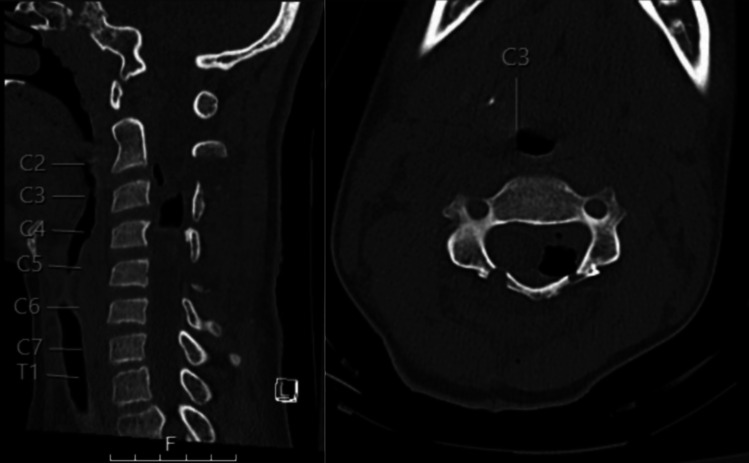
Postoperative non-contrast CT of the cervical spine (sagittal and axial views) displaying gross total resection with expected pneumocephalus and re-expansion of spinal cord within the canal

After brief stabilization and artificial mean arterial pressure (MAP) elevation in the ICU, the patient was taken to the operating room for mass resection. Somatosensory and motor-evoked potentials were monitored and stable throughout the case. A midline incision was performed after localization, and laminectomy was completed from the bottom of C2 to the top of C5. A large, calcified mass was immediately visualized occupying the entirety of the dorsal spinal canal. Under the microscope, we began internally debulking the mass on the left side of the canal, away from the spinal cord. Because the majority of the mass was densely calcified, this was achieved by switching between the high-speed drill and the ultrasonic aspirator. Once all calcified material was removed, the spinal cord began to relax back toward the center of the canal. A capsular, fibrous portion of the tumor remained in the left lateral gutter. We visualized the left C3 and C4 nerve roots, neither of which were directly involved in the lesion. A single arterial pedicle of the lesion that arose from the left exiting C4 nerve root was cauterized and cut, allowing deliverance of the remaining soft tissue mass. There was no CSF leak. A laminoplasty was performed at C3–4. Postoperatively, supraphysiologic MAP goals were maintained for 72 hours. The motor exam improved to nearly full strength in the right upper extremity, with continued improvement in sensation. The patient was discharged home ambulatory on postoperative day 4 (Figs. 1, 2 and 3).

## Discussion

### Histology/Immunohistochemical

**Fig. 4:**
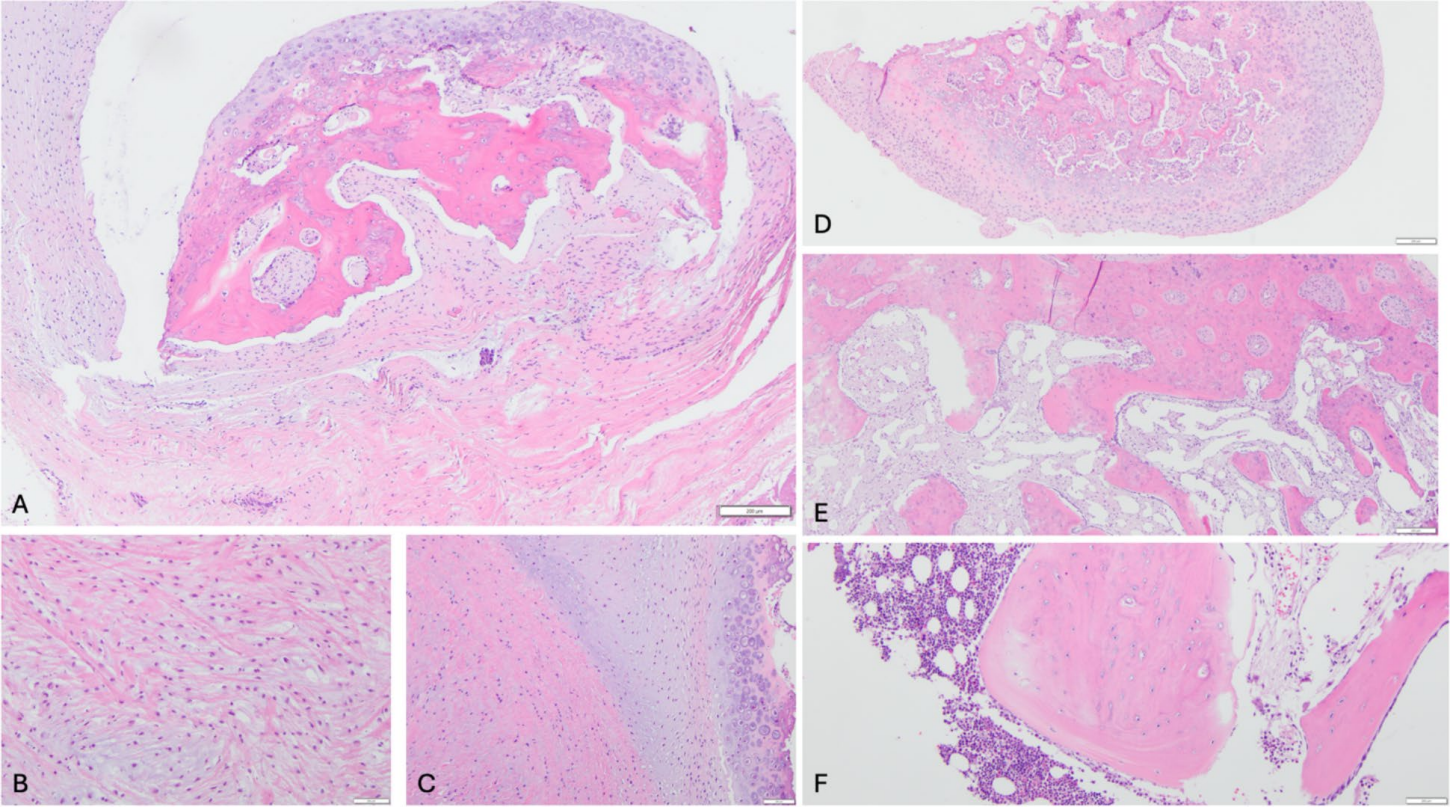
A Low-power view of ligamentous tissue with ossification. B High-power view of fibrocartilage with myxedematous degeneration. C High-power view of transition of degenerating cartilage (left) to endochondral ossification (right). D Low-power view of endochondral ossification. E Trabecular bone and fibrovascular marrow in maturing areas of lesion. F. Trabecular bone and hematopoietic marrow in the most mature foci in the lesion

Histologic evaluation of the mass demonstrated background fibrocartilaginous ligamentous tissue with a myxedematous degeneration (Fig. 4 A and B). There was a zonal transition in the foci of degeneration to hyaline cartilage with subsequent endochondral ossification (Fig. 4 C and D). From these areas, there was a gradual transition to lamellar bony trabeculae with fibrovascular marrow (Fig. 4E). Areas with hematopoietic marrow represented the oldest foci of degeneration within the lesion (Fig. 4).

### Follow-up

Immediate postoperative MRI revealed a gross total resection with re-expansion of the spinal cord. XR at 2 weeks revealed kyphosis at C3–4, which progressed to nearly 50° by 6 weeks postoperatively. The patient subsequently underwent C2–5 anterior cervical discectomy and fusion and has recovered uneventfully continues to demonstrate slowly progressive neurologic improvement.

## Conclusion

While HC can develop after traumatic insult, or as late sequelae of spontaneous hemorrhage or infection, involvement of the cervical spine in a child has not been described. We presented a unique case of HC within the cervical canal in a young patient with acute on chronic myelopathy, initially diagnosed as spastic hemiparesis from cerebral palsy. The inciting event was most likely a perinatal spinal hemorrhage that later calcified, though an epidural abscess following an indolent infection is also possible. In the setting of severe spinal cord compression with motor deficits, complete surgical resection is safe and feasible, and can result in gain of neurologic function despite chronic myelopathic changes. Histological specimens can reveal a zonal arrangement of peripheral spindle cells/fibrous tissue, myxoid/cartilaginous tissue, and an inner core of ossification. This is the first reported pediatric case of HC within the cervical spine presenting acutely as a spinal cord injury, with only two other reported cases of HC within the spine in adults (Table 1) [16, 17]. Close attention should be paid in infancy when there may be an unclear diagnosis for weakness or spasticity without complete neuroaxis imaging.

**Table 1:** Heterotopic calcification case reports – spine

Case report	Age/sex	Location	Final diagnosis
Weerakoon et al. [16]	60F	L4/5 facet	Heterotopic calcification
Bellasri and Asri [17]	35M	S1 nerve root	Neuritis ossificans*

*Neuritis ossificans is described as heterotopic calcification involving nerve roots

## Data Availability

No datasets were generated or analysed during the current study.
